# Clinical and Kinematic Correlates of Favorable Gait Outcomes From Subthalamic Stimulation

**DOI:** 10.3389/fneur.2020.00212

**Published:** 2020-04-22

**Authors:** Idil Cebi, Marlieke Scholten, Alireza Gharabaghi, Daniel Weiss

**Affiliations:** ^1^Department of Neurodegenerative Diseases, Centre for Neurology, Hertie Institute for Clinical Brain Research (HIH), University of Tübingen, Tübingen, Germany; ^2^German Centre of Neurodegenerative Diseases (DZNE), University of Tübingen, Tübingen, Germany; ^3^Tübingen Neuro Campus (TNC), University of Tübingen, Tübingen, Germany; ^4^Division of Functional and Restorative Neurosurgery, Department of Neurosurgery, University of Tübingen, Tübingen, Germany

**Keywords:** deep brain stimulation, subthalamic nucleus, freezing of gait, gait kinematics, Parkinson's disease

## Abstract

**Objective:** Gait and freezing of gait (FoG) are highly relevant to the outcomes of subthalamic nucleus deep brain stimulation (STN-DBS) in Parkinson's disease (PD). Previous studies pointed to variable response to combined dopaminergic and STN-DBS treatment. Here, we performed a prospective exploratory study on associations of preoperative clinical and kinematic gait measures with quantitative gait and FoG outcomes after STN-DBS implantation.

**Methods:** We characterized 18 consecutive PD patients (13 freezers) before and after STN-DBS implantation. The patients received preoperative levodopa challenges (MedOff vs. MedOn) and a postoperative reassessment at 6 months from surgery in MedOn/StimOn condition. We correlated the FoG outcome, calculated as improvement of Freezing of Gait Assessment Course (FoG-AC) from baseline MedOff to 6-month follow-up MedOn/StimOn, with the levodopa response of preoperative clinical and kinematic gait measures. We considered measures with significant correlations for a multiple regression model.

**Results:** We found that the postoperative gait and FoG outcomes were associated with the preoperative levodopa response of clinical and kinematic gait measures. In particular, preoperative levodopa sensitivity of FoG showed high correlation with a favorable quantitative FoG outcome. Among kinematic measures, preoperative levodopa response of stride length and range of motion showed high correlation with favorable FoG outcome. In addition, the preoperative levodopa sensitivity of FoG predicted postoperative FoG outcome with high accuracy (*R*^2^ = 0.952; 95% CI: 0.95–1.29; *P* < 0.001).

**Conclusions:** Preoperative clinical and kinematic measures correlated with favorable postoperative gait and FoG outcomes. The findings should be reproduced in larger and independent cohorts to verify their predictive value.

## Introduction

Gait disturbance and freezing of gait (FoG) hamper quality of life and lead to falls and morbidity in Parkinson's disease (PD) (1, 2). Given the variable response to dopaminergic medication and deep brain stimulation of the subthalamic nucleus (STN-DBS), a preoperative stratification of postoperative gait and FoG outcomes is needed (3–6). Because gait and FoG outcomes did not constitute primary endpoints in previous large-scale, randomized controlled trials, the present evidence is incomplete (7, 8). One recent meta-analysis suggested that the levodopa response of the Movement Disorder Society (MDS)-sponsored Revision of the Unified Parkinson's Disease Rating Scale part III (MDS-UPDRS III) summary score indicates favorable FoG outcome (9). A recent secondary analysis of the EARLYSTIM cohort found that the patients with preoperative FoG were more likely to stay freezers at 24-month follow-up when having longer disease duration (10).

To improve therapeutic decisions in the context of FoG, fine-grained multimodal measures specific to gait and FoG outcomes are needed to account for the complexity of postoperative outcomes of gait. Beyond clinical variables, sensor-based kinematic features receive converging interest in order to achieve objective and quantitative features of PD gait (11, 12). Some parameters such as reduction in step length, decreased velocity, increased cadence, stride-to-stride variability (13), and lower limb asymmetry (14) are precursors for FoG and falls (15). Previous studies concluded that both dopaminergic medication and STN-DBS modulated such features alone or in combination (16–20), in particular, stride length, velocity, and range of motion (ROM) at hip, knee, and ankle levels (16–19, 21–23).

In this study, we characterized consecutive patients with idiopathic PD evaluated for STN-DBS therapy with respect to gait measures, and FoG in particular, including clinical and kinematic gait and FoG assessments. First, patients received a diagnostic workup of gait measures in a preoperative levodopa challenge. Then, patients were followed up postoperatively, and both (i) the effect of STN-DBS 8 weeks after surgery and (ii) the overall gait and FoG outcome of combined dopaminergic medication and STN-DBS 6 months after surgery were characterized. The aim of this prospective study was to explore meaningful clinical and kinematic candidate features that correlate with favorable gait and, in specific, quantitative FoG outcomes. With this study, we wished to identify candidate features for a future larger and independent prospective prediction study.

## Materials and Methods

The trial was approved by the Ethics Committee of the University of Tübingen (355/2015BO1). All patients participated with written informed consent.

### Patients

We consecutively recruited 24 advanced PD patients among DBS candidates during an inpatient-screening visit between May 2015 and November 2016. Inclusion criteria were disease duration longer than 5 years and age >18 and <80 years. The presence of FoG was not an inclusion criterion. Exclusion criteria were as follows: cognitive impairment [Mini-Mental State Examination (MMSE) <25], participation in other clinical trials, and chronic pathological conditions interfering with the interpretability of gait assessments (e.g., major orthopedic or psychiatric conditions like depression or psychosis).

Out of the 24 patients, 18 underwent subthalamic nucleus implantation (STN-DBS) according to regular DBS indication criteria (7, 8). The remaining six patients stayed under best oral medical treatment. Reasons were sufficient amelioration of tremor (*n* = 2), sufficient amelioration of motor fluctuations (*n* = 3) after optimizing oral medication, or lack of objective motor fluctuations (*n* = 1) (8, 24).

The mean age of the study cohort (*n* = 18) was 66.9 ± 6.9 years, and mean disease duration was 12.8 ± 6.0 years. The median MMSE score was 30 [min 26–max 30]. The levodopa equivalent dosage at baseline was 1,334 ± 147 mg/day. Detailed patient characteristics are given in Table 1.

**Table 1:** Patient characteristics at baseline.

**ID**	**Age as range**	**Disease duration (years)**	**LEDD (mg/d)**	**MMSE**	**MDS-UPDRS III MedOff**	**MDS-UPDRS III MedOn**	**MDS-UPDRS I**	**MDS-UPDRS II**	**MDS-UPDRS IV**	**H&Y stage MedOff**	**DBS indication**	**Freezing of gait**
2	50–59	7	813	30	51	22	8	10	0	2	2	0
3	70–79	17	821	28	67	48	7	31	10	4	1	1
4	70–79	5	0*	26	40	29	12	15	0	2	2	0
5	70–79	10	2,281	30	71	39	3	27	3	4	1	1
6	70–79	15	1,830	30	40	12	11	25	5	4	1	1
9	60–69	9	1,198	30	22	15	8	10	6	3	1	1
11	70–79	12	1,460	30	55	26	16	11	7	4	1	1
12	50–59	10	2,069	29	56	22	11	26	5	3	1	1
14	60–69	24	2,081	29	53	29	15	25	10	3	1	1
15	60–69	8	1,633	28	57	34	11	26	12	4	1	1
16	60–69	20	833	30	76	45	16	13	8	4	1	1
17	60–69	11	800	30	44	30	3	13	3	2	2	0
18	60–69	13	1,158	30	38	17	13	4	6	2	1	0
19	70–79	5	1,198	30	45	32	7	13	4	3	1	1
20	60–69	13	1,221	30	28	10	9	6	5	3	1	1
21	70–79	27	2,438	30	38	28	12	24	15	3	1	0
22	70–79	13	981	30	36	19	7	15	8	3	1	1
23	50–59	11	1,200	29	43	18	16	18	13	3	1	1

*IDs 1, 7, 8, 10, 13, and 24 (not listed) have not been identified with relevant motor fluctuations or therapy-resistant tremor during DBS screening and therefore continued best medical treatment. Gender: m, male; f, female. LEDD, l-Dopa equivalent daily dosage; MMSE, Mini-Mental State Examination; H&Y, Hoehn and Yahr. DBS indication: 1 = motor fluctuations and 2 = therapy-resistant tremor. Freezing of gait: 0 = non-freezer and 1 = definite freezer. We classified the patients by performing the Freezing Assessment Course of Ziegler and colleagues and by using the Freezer classification algorithm of Snijders and colleagues*.

* *Patient had therapy-resistant tremor*.

### Study Design

Patients underwent a preoperative “baseline assessment” in two conditions: (i) clinical off-state after overnight withdrawal of dopaminergic medication (MedOff) and (ii) clinical on-state (MedOn) assessed within 1 h after intake of immediate release levodopa preparation (1.5-fold individual morning dose). We performed (i) a 7-m timed walking test [Core Assessment Program for Surgical Interventional Therapies in Parkinson's Disease (CAPSIT-PD)], (ii) Freezing of Gait Assessment Course (FoG-AC) for quantitative FoG assessment (25), and (iii) Push and Release Test for assessing postural stability (26). These tests were performed using wearable inertial measurement units (APDM Inc., Portland, OR, USA). Additional clinical characterization included MDS-UPDRS III, Postural Instability and Gait Disorder (PIGD) subscore (sum of items 10–12 from MDS-UPDRS III), and Berg Balance Scale. For the PIGD subscore, we decided to use only clinical motor items 3.10–3.12 from MDS-UPDRS part III. We did not use the MDS-UPDRS II items 2.12 and 2.13, which rely on patient reporting. The main reason for this decision was that we expected MDS-UPDRS II not to be sensitive to the clinical transitions between clinical conditions (i.e., narrow time intervals between preoperative MedOff vs. MedOn and postoperative StimOff vs. StimOn). We further obtained MDS-UPDRS parts I, II, and IV and Parkinson's Disease Questionnaire (PDQ-39) for quality of life.

Eighteen patients underwent surgery for bilateral STN-DBS with Medtronic quadripolar leads, model 3389 (*n* = 17) or model 3387 (*n* = 1); and all received an Activa PC impulse generator (Medtronic, Minneapolis, Minnesota). No surgical complications were reported. DBS was turned on within the first week after surgery. Three patients experienced falls in the first 8 weeks after surgery (ID 9 owing to an accident; ID 12 and 20 owing to FoG). One patient required inpatient care and surgery owing to radius fracture. One patient showed psychosis (ID 15) 10 weeks after surgery, which reverted under treatment with clozapine 25 mg/day. An “interim assessment” with a detailed reprogramming session was carried out 8 weeks from surgery after attenuation of the postoperative stun effect (i) to achieve optimized stimulation settings and (ii) to determine the pure STN-DBS effect on gait and FoG. This reprogramming session was performed after overnight withdrawal of all dopaminergic medication in MedOff/StimOff and MedOff/StimOn conditions. We performed first MedOff/StimOff assessment about 30 min after switching the stimulation off and then MedOff/StimOn assessment at least 30 min after switching the stimulation on. We used a similar approach elsewhere (27). We performed the CAPSIT-PD, the FoG-AC, the Push and Release Test, and MDS-UPDRS III in both conditions.

**Figure 1:**
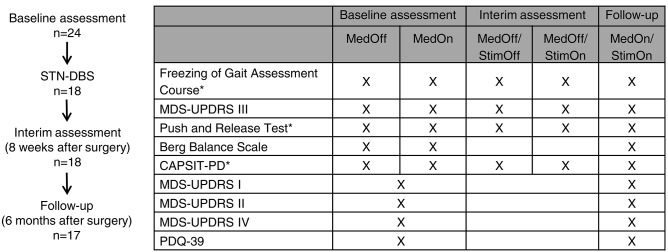
Study design and protocol. *Tests were performed using wearable inertial measurement units (APDM Inc., Portland, OR, USA).

A detailed postoperative characterization was performed 6 months from implantation under the best individual treatment (follow-up MedOn/StimOn). Detailed information on the stimulation parameters is provided (Table e-1). One patient was lost to follow-up after retracting consent (ID 2). We decided for this time point to ensure that the postoperative stun effect would have fully remitted and to ensure that stimulation parameters and medication had been adjusted. We decided for the MedOn/StimOn condition, because we wished to determine the gait and FoG outcome in the treatment state close to the regular daily life conditions. Furthermore, we reasoned that another MedOff session and reinsertion of levodopa by means of a challenge would not have reflected the true daily life outcome. However, this choice also meant that we assessed the pure stimulation effect at the 8-week interim assessment, but not at the postoperative 6-month follow-up. The detailed study protocol is given in Figure 1.

### Kinematic Recordings

Three wearable Opal® inertial measurement units (APDM Inc., Portland, OR, USA) were used for gait kinematic analysis. These sensors comprise tri-axial accelerometer, gyroscope, and magnetometer attached to both ankles and in lumbar position. We computed the kinematic gait measures with the “Mobility Lab” algorithm (APDM Inc., Portland, OR, USA) (28). The spatial (stride length), temporal (gait cycle time), and spatiotemporal (stride velocity) parameters as well as ROM of shanks and knees were extracted. In addition, we computed swing time asymmetry (Equation 1).

(1) $$\begin{array}{r} \text{Swing time asymmetry}=100\;\times \frac{\left|\text{SWTleft }-\text{ SWTright}\right|}{max\left(\left[\text{SWTleft},\text{ SWTright}\right]\right)} \end{array}$$

In two patients, the quality of the kinematic time series was not adequate for analysis owing to technical problems (ID 3 at follow-up; ID 16 at baseline MedOff).

### Outcome Measures and Statistical Analysis

In this prospective study, we aimed to explore the preoperative clinical and kinematic correlates from a comprehensive set of candidate features potentially related to gait and FoG outcomes.

#### Quantitative Comparisons of Outcomes Including Levodopa and Stimulation Effects on the Entire Group and on the Freezer Subgroup

We first assessed the preoperative levodopa response (MedOff vs. MedOn) at “baseline assessment” on clinical gait, FoG, balance scores, and kinematic measures. Then, we defined the stimulation effect by comparing MedOff/StimOff and MedOff/StimOn conditions during the “interim assessment,” which took place 8 weeks from surgery. A comparison of MedOff condition from “baseline assessment” and MedOn/StimOn condition from “follow-up” determines the gait and FoG outcomes. We tested for normal distribution using the Kolmogorov–Smirnov test (*P* < 0.05). To compare between conditions, we used *t*-test for parametric data, Wilcoxon, or sign test for non-parametric data. We performed the analysis for the entire group and for the freezer subgroup. Patients were classified as definite freezers according to previous criteria (29), that is, if the examiner observed FoG. We corrected the clinical measures as well as the kinematic measures using the false discovery rate (FDR) (30).

#### Correlations of Preoperative Scores With Postoperative 6-Month Freezing Outcome in the Freezer Subgroup

We tested whether preoperative clinical and kinematic variables with significant levodopa response correlate with the postoperative 6 months outcome of regular therapy in MedOn/StimOn. We used Spearman correlations to this end.

#### Prediction of the 6-Month Freezing Outcome From Preoperative Clinical and Kinematic Variables in the Freezer Subgroup

Owing to the limited sample size of this monocentric study, we did not design the study as a prediction study. However, following an exploratory approach, we evaluated whether valid predictions of the FoG outcomes in terms of FoG-AC (as dependent variable, calculated as improvement of FoG-AC from baseline MedOff to 6-month follow-up MedOn/StimOn) could be made from the preoperative levodopa-sensitive clinical and kinematic measures. This means that we carried forward the preoperative variables (MedOn–MedOff) that showed significant correlation with the postoperative 6 months outcomes of FoG. We considered these findings rather hypothesis-generating instead of confirmatory, owing to the limited sample size and the high selection in this cohort; that is, the findings should be reproduced in independent cohorts. We used a stepwise multiple regression model to this end.

All statistical analyses were performed with IBM SPSS statistics, version 25.0 (IBM Deutschland GmbH, Ehningen, Germany). We report descriptive statistics as mean ± SD for parametric data and median [min–max] for non-parametric data depending on their distribution.

## Results

### Comparison of Gait Outcomes on the Entire Group

A detailed overview of the statistical descriptives and results of the “entire group” analysis is given in Table 2.

**Table 2:** Clinical and kinematic data of entire group.

	**Baseline MedOff**	**Baseline MedOn**	**Interim MedOff/StimOff**	**Interim MedOff/StimOn**	**Follow-up (FU) MedOn/StimOn**	***P*-value Baseline Off vs. FU**	***P*-value Baseline Off vs. On**	***P*-value Interim Off vs. On**
MDS-UPDRS III^c^	47.78 ± 14.35	26.39 ± 10.71	50.39 ± 16.43	33.33 ± 11.11	28.88 ± 11.94	<0.001*	<0.001*	<0.001*
PIGD subscore^c^	5.44 ± 2.92	2.39 ± 2.09	4.61 ± 2.89	3.22 ± 2.58	2.82 ± 2.74	0.002*	<0.001*	0.006*
Push and release Test^a^	1 [0–4]	1 [0–4]	1 [0–4]	1 [0–4]	0 [0–4]	0.013*	0.070	1.000
Berg balance scale^b^	43 [9-56]	55 [10-56]			56 [11-56]	0.013*	0.001*	
CAPSIT-PD time^b^	28 [11-533]	16 [9-42]	23.5 [12-360]	15 [9-93]	15 [11-257]	0.017*	0.001*	0.002*
CAPSIT-PD steps^b^	54 [18-500]	26 [18-65]	42.5 [21-500]	29.5 [20-175]	27 [18-330]	0.009*	0.001*	0.004*
ROM shank^b^	37.63 [10.10–74.12]	66.99 [26.69–81.71]	48.22 [11.10–79.21]	62.11 [20.08–78.48]	67.41 [14.56–80.04]	0.008*	<0.001*	0.006*
ROM knee^b^	38.05 [16.30–53.44]	49.20 [26.76–56.13]	42.01 [20.31–52.68]	47.38 [26.71–55.49]	49.12 [23.55–59.25]	0.005*	0.003*	<0.001*
Mean stride length^b^	42.38 [11.22–80.43]	73.30 [25.92–85.22]	47.21 [12.36–84.63]	61.06 [21.22–80.82]	69.60 [13.60–88.03]	0.012*	0.001*	0.028
Mean stride velocity^b^	41.20 [7.17–84.65]	62.27 [23.31–89.80]	47.44 [9.97–81.28]	59.67 [21.83–84.97]	64.93 [18.76–79.62]	0.078	0.003*	0.053
Mean gait cycle time^b^	1.09 [0.64–1.65]	1.12 [0.94–1.35]	1.14 [0.59–1.45]	1.05 [0.88–1.36]	1.09 [0.85–1.35]	0.955	0.687	0.500
Swing time asymmetry (STA)^b^	3.80 [0.23–40.57]	7.23 [0.30–34.82]	7.15 [0.24–47.37]	7.17 [0.92–50.31]	8.02 [1.09–41.49]	0.041	0.687	0.679

*PIGD, Postural Instability and Gait Disorder subscore (sum of items 10–12 from MDS-UPDRS III); CAPSIT-PD, 7-m timed walking test from Core Assessment Program for Surgical Interventional Therapies in Parkinson's Disease; ROM, range of motion. Baseline: preoperative assessment. Interim: postoperative assessment 8 weeks from surgery. Follow-up: postoperative assessment 6 months from surgery. Values are described as mean ± SD or median [min–max]. Two-sided P-values are given. One patient (ID 23) was not able to complete CAPSIT-PD at baseline MedOff condition. The kinematic data quality of ID 16 at baseline MedOff and ID 3 at follow-up was not adequate for analysis*.

a *Sign test*.

b *Wilcoxon signed-rank test*.

c *Paired sample t-test*.

* *Significant after false discovery rate (FDR) correction (Benjamini–Hochberg)*.

#### Preoperative MedOff vs. MedOn

MedOn improved the CAPSIT-PD in “number of steps” (*P* = 0.001) and “time” (*P* = 0.001). We observed an improvement of joint ROM at shank level (*P* < 0.001) and knee level (*P* = 0.003), of stride length (*P* = 0.001), and stride velocity (*P* = 0.003). There was no difference in gait cycle time and swing time asymmetry.

#### 8-Week StimOff vs. StimOn

CAPSIT-PD showed an improvement with STN-DBS in the “number of steps” (*P* = 0.004) and “time” (*P* = 0.002). Spatiotemporal and kinematic gait parameters showed an improvement of joint ROM at shank level (*P* = 0.006) and at knee level (*P* < 0.001) and of stride length (*P* = 0.028, n.s. after FDR correction). There was no difference in stride velocity, gait cycle time, and swing time asymmetry.

#### Preoperative MedOff vs. 6-Month MedOn/StimOn

CAPSIT-PD showed an improvement 6 months after STN-DBS implantation in the “number of steps” (*P* = 0.009) and “time” (*P* = 0.017). We observed an improvement of joint ROM at shank level (*P* = 0.008) and knee level (*P* = 0.005) and of stride length (*P* = 0.012). There was no difference in stride velocity and gait cycle time. Swing time asymmetry worsened after 6 months (*P* = 0.041, n.s. after FDR correction).

### Comparison of Gait and Freezing of Gait Outcomes in the Freezer Subgroup

**Figure 2:**
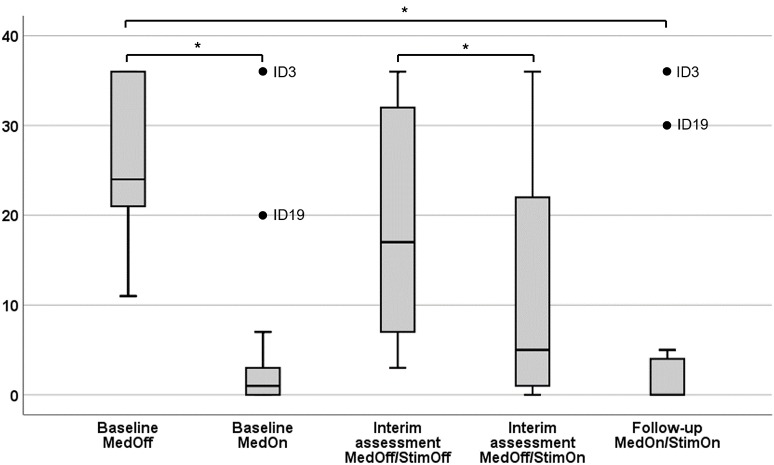
Severity of Freezing Assessment Course in different conditions. Results are given as box plots. *x*-axis, therapeutic condition; *y*-axis, score of the Freezing of Gait Assessment Course. *Significant after false discovery rate (FDR) correction (Benjamini–Hochberg).

A detailed overview of the statistical descriptives and results of “freezer subgroup” analysis is given in Table 3. The FoG-AC scores at each assessment and condition are given in Figure 2 as box plots.

**Table 3:** Clinical and kinematic data of freezing patients.

	**Baseline MedOff**	**Baseline MedOn**	**Interim MedOff/StimOff**	**Interim MedOff/StimOn**	**Follow-up (FU)**	***P*-value Baseline Off vs. FU**	***P*-value Baseline Off vs. On**	***P*-value Interim Off vs. On**
FoG-AC^b^	24 [11-36]	1 [0–36]	17 [3-36]	5 [0–36]	0 [0–36]	0.003*	0.002*	0.003*
MDS-UPDRS III^c^	49.92 ± 16.24	26.85 ± 12.30	51.08 ± 17.14	34.15 ± 12.11	31.08 ± 11.54	<0.001*	<0.001*	<0.001*
PIGD subscore^c^	6.77 ± 2.05	2.62 ± 1.98	5.31 ± 2.72	3.69 ± 2.59	2.85 ± 2.73	<0.001*	<0.001*	0.019*
Push and release test^a^	2 [0–4]	1 [0–4]	1 [0–3]	1 [0–3]	0 [0–3]	0.012*	0.125	1.000
Berg balance scale^b^	42 [9-56]	53 [10-56]			55 [11-56]	0.019*	0.003*	
CAPSIT-PD time^b^	38.5 [12-533]	16 [9-42]	29 [13-360]	16 [9-93]	15 [11-257]	0.041*	0.002*	0.006*
CAPSIT-PD steps^b^	62.5 [25-500]	27 [21-65]	54 [26-500]	36 [24-175]	27 [22-330]	0.028*	0.002*	0.017*
CAPSIT-PD freezing^b^	0 [0–31]	0 [0–0]	0 [0–9]	0 [0–8]	0 [0–43]	0.715	0.180	0.046
ROM shank^b^	36.63 [10.10–74.12]	66.06 [26.68–78.95]	40.61 [11.10–71.86]	53.77 [20.08–74.32]	67.42 [14.56–79.69]	0.010*	0.002*	0.009*
ROM knee ^b^	34.19 [16.30–51.25]	49.05 [26.76–54.04]	39.48 [20.31–49.05]	44.82 [26.71–52.81]	49.12 [23.55–59.25]	0.010*	0.002*	0.002*
Mean stride length^b^	39.78 [11.22–80.43]	69.96 [25.92–84.66]	43.37 [12.36–77.10]	57.78 [21.22–77.64]	69.69 [13.60–88.04]	0.013*	0.002*	0.028
Mean stride velocity^b^	34.58 [7.17–84.65]	61.10 [23.31–89.80]	47.82 [9.97–81.28]	57.47 [23.50–79.67]	64.93 [18.76–79.62]	0.033*	0.008*	0.101
Mean gait cycle time^b^	1.05 [0.64–1.65]	1.15 [0.94–1.35]	1.07 [0.59–1.45]	1.01 [0.88–1.36]	1.08 [0.85–1.26]	0.594	0.937	0.507
Swing time asymmetry (STA)^b^	4.42 [0.23–40.57]	6.68 [0.30–34.82]	6.99 [0.31–47.37]	10.11 [0.92–50.31]	8.03 [1.09–41.49]	0.213	0.530	0.249

*FoG-AC, Freezing of Gait Assessment Course (Ziegler and colleagues); PIGD, Postural Instability and Gait Disorder subscore (sum of items 10–12 from MDS-UPDRS III); CAPSIT-PD, 7-m timed walking test from Core Assessment Program for Surgical Interventional Therapies in Parkinson's Disease; ROM, range of motion. Baseline: preoperative assessment. Interim: postoperative assessment 8 weeks from surgery. Follow-up: postoperative assessment 6-months from surgery. Values are described as mean ± SD or median [min–max]. Two-sided P-values are given. One patient (ID 23) was not able to complete CAPSIT-PD at baseline MedOff condition. The kinematic data quality of ID 16 at baseline MedOff and ID 3 at follow-up was not adequate for analysis*.

a *Sign test*.

b *Wilcoxon signed-rank test*.

c *Paired sample t-test*.

* *Significant after false discovery rate (FDR) correction (Benjamini–Hochberg)*.

#### Preoperative MedOff vs. MedOn

FoG-AC improved (*P* = 0.002) preoperatively, as well as the CAPSIT-PD in the “number of steps” (*P* = 0.002) and “time” (*P* = 0.002). Spatiotemporal and kinematic gait parameters showed an improvement of ROM at shank level (*P* = 0.002) and at knee level (*P* = 0.002) as well as stride length (*P* = 0.002) and stride velocity (*P* = 0.008). There was no difference in gait cycle time and swing time asymmetry.

#### 8-Week StimOff vs. StimOn

With STN-DBS turned on, the FoG-AC improved (*P* = 0.003). CAPSIT-PD showed also an improvement in “number of steps” (*P* = 0.017), “time” (*P* = 0.006), and “number of freezing episodes” (*P* = 0.046, n.s. after FDR correction). Spatiotemporal and kinematic gait parameters showed an improvement of joint ROM at shank level (*P* = 0.009) and knee level (*P* = 0.002) and of stride length (*P* = 0.028, n.s. after FDR correction). There was no difference in stride velocity, gait cycle time, and swing time asymmetry.

#### Preoperative MedOff vs. 6-Month MedOn/StimOn

FoG-AC improved between postoperative 6-month follow-up MedOn/StimOn and baseline MedOff (*P* = 0.003). CAPSIT-PD showed improvements in the “number of steps” (*P* = 0.028) and “time” (*P* = 0.041). We observed an improvement of joint ROM at shank level (*P* = 0.010) and knee level (*P* = 0.010), of stride length (*P* = 0.013), and of stride velocity (*P* = 0.033). There was no difference in gait cycle time and swing time asymmetry.

### Correlations of Preoperative Scores With Postoperative 6-Month Freezing Outcome in the Freezer Subgroup

We observed that a favorable outcome of FoG at 6-month follow-up (calculated as improvement of FoG-AC from baseline MedOff to 6-month follow-up MedOn/StimOn) correlated with preoperative severity of FoG in MedOff (defined by FoG-AC score) (*P* = 0.016), as well as with preoperative levodopa response of the FoG-AC (*P* < 0.001) and the preoperative levodopa response of the PIGD subscore (*P* = 0.004).

**Figure 3:**
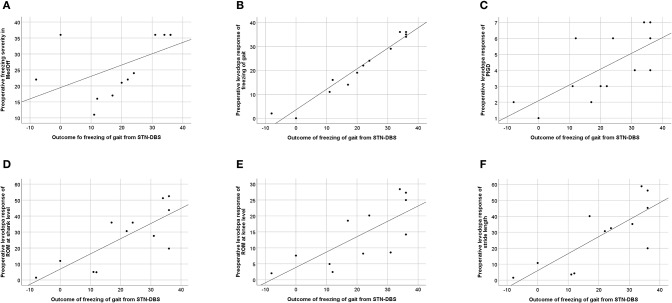
Correlation between freezing of gait (FoG) outcome, calculated as improvement of Freezing of Gait Assessment Course (FoG-AC) from baseline MedOff to follow-up MedOn/StimOn. **(A)** Preoperative FoG severity in baseline MedOff, defined by FoG-AC score (*r* = 0.649; *P* = 0.016). **(B)** Preoperative levodopa response of freezing of gait, calculated as improvement of FoG-AC from baseline MedOff to baseline MedOn (*r* = 0.957; *P* < 0.001). **(C)** Preoperative levodopa response of PIGD subscore, calculated as improvement of PIGD subscore from baseline MedOff to baseline MedOn (*r* = 0.743; *P* = 0.004). **(D)** Preoperative levodopa response of ROM at shank level, calculated as improvement of ROM at shank level from baseline MedOff to baseline MedOn (*r* = 0.746; *P* = 0.005). **(E)** Preoperative levodopa response of ROM at knee level, calculated as improvement of ROM at knee level from baseline MedOff to baseline MedOn (*r* = 0.817; *P* = 0.001). **(F)** Preoperative levodopa response of stride length, calculated as improvement of stride length from baseline MedOff to baseline MedOn (*r* = 0.761; *P* = 0.004).

Among kinematic parameters, preoperative levodopa response of stride length (*P* = 0.004), ROM at shank (*P* = 0.005), and ROM at knee level (*P* = 0.001) pointed to a favorable outcome of FoG. All correlations are given in Table 4, and the significant correlations can be found in Figure 3.

**Table 4:** Clinical and kinematic variables correlating to favorable FoG outcome.

	**Correlation coefficient**	***P*-value**	***N***
Preoperative LEDD	0.459	0.115	13
Age	0.177	0.562	13
Disease duration	0.202	0.508	13
Preoperative severity of UPDRS III in MedOff	−0.028	0.929	13
Preoperative severity of FoG in MedOff	0.649	0.016*	13
Preoperative levodopa response of FoG	0.957	<0.001*	13
Preoperative levodopa response of PIGD subscore	0.743	0.004*	13
Preoperative levodopa response of UPDRS III	0.425	0.147	13
Preoperative levodopa response of ROM shank	0.746	0.005*	12
Preoperative levodopa response of ROM knee	0.817	0.001*	12
Preoperative levodopa response of stride length	0.761	0.004*	12
Preoperative levodopa response of stride velocity	0.394	0.205	12
Preoperative levodopa response of gait cycle time	−0.113	0.727	12
Preoperative levodopa response of swing time asymmetry	0.458	0.135	12

*FoG-AC, Freezing of Gait Assessment Course (Ziegler and colleagues); PIGD, Postural Instability and Gait Disorder subscore (sum of items 10–12 from MDS-UPDRS III); ROM, range of motion. Two-sided P-values are given*.

* *Significant after false discovery rate (FDR) correction (Benjamini–Hochberg)*.

### Prediction of the 6-Month Follow-Up Freezing Outcome From Preoperative Clinical and Kinematic Variables in the Freezer Subgroup

We included the clinical and kinematic variables to our regression model that showed significant correlation with FoG-AC outcomes, namely, the preoperative levodopa response of FoG-AC, preoperative levodopa response of PIGD subscore, preoperative levodopa response of ROM shank, preoperative levodopa response of ROM knee, preoperative levodopa response of stride velocity, and preoperative levodopa response of stride length. The preoperative levodopa response of FoG-AC predicted the postoperative outcome (difference between 6-month follow-up in MedOn/StimOn condition and preoperative MedOff condition) to a great extent (*R*^2^ = 0.952, 95% CI: 0.95–1.29, *P* < 0.001) (Figure 3B). Levodopa response of the other variables did not show significant predictive values (preoperative levodopa response of PIGD subscore *P* = 0.086, preoperative levodopa response of ROM at shank *P* = 0.508, preoperative levodopa response of ROM at knee *P* = 0.666, preoperative levodopa response of stride velocity *P* = 0.959, and preoperative levodopa response of stride length *P* = 0.200). In addition, these kinematic variables did not improve the accuracy of our prediction model.

## Discussion

In this prospective study, we found that both clinical and kinematic levodopa responsive gait measures improved after STN-DBS. This was in particular true for the quantitative FoG outcomes in PD freezers. Interestingly, the preoperative response assessed with the fine-grained clinical FoG Assessment Course and the kinematic gait features were associated with a favorable outcome of FoG.

These findings are relevant to improve the preoperative stratification and patient counseling on STN-DBS procedures with respect to gait and FoG. We identified the preoperative severity of FoG in medication off state, as well as the preoperative levodopa response of quantitative FoG assessment and preoperative levodopa response of PIGD subscore as candidate features related to a favorable FoG outcome. Likewise, preoperative levodopa response of ROM at shank and knee levels and that of stride length were also such candidate features among kinematic measures. Previous studies suggested that gait outcomes after STN-DBS are highly heterogeneous with up to 50% of preoperative “freezers” continuing FoG 1 year after surgery (3) and 42% in another study (4). Furthermore, FoG seems to worsen substantially under STN-DBS as disease progresses, and reprogramming strategies like low-frequency programming of STN stimulation or interleaved stimulation of STN+SNr may lead to partial success (6, 27, 31). However, the long-term effects of such reprogramming are variable, and definite conclusion cannot be drawn yet on these strategies (27, 31). Together, the preoperative stratification process needs refinement in order to identify the patients with favorable gait and FoG outcomes (32, 33).

In this sense, one recent meta-analysis found the preoperative levodopa response of the total UPDRS III to predict favorable FoG outcomes on the UPDRS II item 14 on FoG (which reflects narrative information on FoG on the basis of the patients' self-perception) (9). In addition, a recent secondary analysis of the EARLYSTIM-trial showed that 52% of the patients had FoG preoperatively, and it decreased to 34% at 24-month follow-up. Interestingly, patients who stayed as freezers had significantly longer disease duration than had those who became non-freezers (10). As main difference to our study, the EARLYSTIM cohort was younger than our cohort (mean 52.6 ± 6.3 years) and had a shorter disease duration (mean 7.5 ± 2.8 years) in comparison with our study (mean age 66.9 ± 6.9 years and mean disease duration 12.8 ± 6.8 years). Further, FoG assessment was based on patient-reported FoG in terms of item 14 of UPDRS II, whereas our study used a quantitative clinical assessment specific to gait as outcome parameter. Similarity between the two studies is a well-preserved levodopa-response of motor symptoms, which is established as a main selection criterion for DBS (7, 8, 34). This study, as well as related studies, increasingly support that the importance of levodopa sensitivity also applies for FoG.

However, given that some patients continue to exhibit FoG despite good levodopa response, more specific quantitative outcomes and predictors on gait measures and FoG in specific are needed. Interestingly, the preoperative correlates identified in this study include FoG severity as clinical measure and also spatial kinematic measures like ROM and stride length. This is in good accordance with previous literature that showed modulation of spatial parameters with STN-DBS (21, 35). In contrast, we did not find limb asymmetry measures to inform about the 6 months FoG outcomes. This is an important caveat, because acute reprogramming strategies in STN-DBS therapy aim to improve lower limb asymmetry measures in order improve FoG outcomes. But also with STN-DBS reprogramming, asymmetry seems to be a short-term marker (36), whereas its role for stable long-term outcomes is less clear and under ongoing consideration (37). Therefore, the correlates of a stable 6 months gait outcome may be more relevant to overall FoG outcomes, as opposed to short-term neuromodulation effects on gait.

### Methodological Considerations and Study Limitations

This study, in which we aimed to identify correlates of 6 months gait and FoG outcomes in patients treated with STN-DBS, was performed with exploratory intent. Because of this and the fact that this was a single-center study, the sample size was limited. Nevertheless, we focused on narrowing down the clinical and kinematic features that correlated with favorable FoG outcomes. Surely, this is not a final confirmation of these findings. Hence, the prediction model—albeit significant—should be reproduced in a larger cohort. The data from this prospective study provide a good basis to challenge the measures in a larger prediction study.

In this study, FoG outcomes were primarily predicted from the preoperative FoG-AC scores. The kinematic measures did not further improve the prediction accuracy of our model; this should be re-challenged in the future. In particular, our patients showed near-to-full levodopa response of FoG preoperatively. However, this must not necessarily be true in other patients with motor fluctuations referred to STN-DBS. In addition to FoG-AC assessments, other clinical measures of long-term response of FoG should be included, like FoG questionnaire. Eventually, in the future, ambulatory kinematic measurements may enable objective assessments of FoG under daily life conditions to get even superior information on the true outcomes of a highly episodic and context-dependent clinical symptom (11).

Patients with unfavorable gait and FoG outcomes after STN-DBS may have been under-represented in this study given that we found a high rate of responders on gait and FoG measures. This may have been caused by the fact that the PD patients were selected according to existing clinical criteria, with levodopa-responsive parkinsonism being one core component in the selection process. It was not intended in this study to refer patients outside these stringent criteria for STN-DBS.

The fact that we assessed only MedOn/StimOn condition at follow-up may be seen as a limitation on the one hand, but on the other hand, this therapeutic condition is closest to the patients' daily life situation and, therefore, most representative for the true therapeutic outcome. Withdrawal and reinsertion of levodopa at the 6-month follow-up would have enabled the evaluation of pure stimulation effect. However, it would have been less representative for the true daily life outcome, because of the artificial condition induced by reinsertion of immediate release levodopa. This was the main reason for our choice.

## Conclusion

In summary, our findings show that favorable outcomes on gait and FoG from STN-DBS are achieved if quantitative FoG measures, stride length, and ROM at shank and knee levels show robust preoperative levodopa response. This study provides novel candidate features specific to gait and FoG that should be re-evaluated and reproduced as predictive stratification model in order to facilitate gait outcomes in STN-DBS for PD.

## Data Availability Statement

The datasets generated for this study are available on request to the corresponding author.

## Ethics Statement

The studies involving human participants were reviewed and approved by University of Tübingen. The patients/participants provided their written informed consent to participate in this study.

## Author Contributions

IC, AG, and DW designed and conceptualized the study. IC, MS, and DW analyzed and interpreted data. IC, MS, AG, and DW drafted and revised the manuscript for intellectual content.

## Conflict of Interest

IC, MS, AG report no disclosures relative to the research covered in the submitted manuscript. DW received research grants from the German Research Council (DFG, WE5375/1-1, WE5375/1-3) and research funding from Medtronic, as well as speakers honoraria/travel grants from Medtronic, Abott (St. Jude), Boston Scientific, and Abbvie.
