# Supplementary material for: Clinical and Kinematic Correlates of Favorable Gait Outcomes From Subthalamic Stimulation
Source: Front Neurol. 2020 Apr 22;11:212. doi: 10.3389/fneur.2020.00212 (PMC7213078; doi:10.3389/fneur.2020.00212)
Supplement: Supplementary file 1 [file Table_1.DOCX]

**Table e-1:** Stimulation parameters at follow-up

| **ID** | **Contacts** | **Amplitude (V)** | **Pulse width (µs)** | **Frequency (Hz)** | **Contacts** | **Amplitude (V)** | **Pulse width (µs)** | **Frequency (Hz)** |
| --- | --- | --- | --- | --- | --- | --- | --- | --- |
| 2 | lost to follow-up | | | | | | | |
| 3 | 2- C+ | 3.5 | 60 | 130 | 10- C+ | 3.2 | 60 | 130 |
| 4 | 2- C+ | 3.1 | 60 | 130 | 10- 11+ | 4.2 | 60 | 130 |
| 5 | 2- C+ | 3.3 | 60 | 130 | 10- C+ | 3.7 | 60 | 130 |
| 6 | 2- C+ | 1.7 | 60 | 130 | 10- C+ | 1.5 | 60 | 130 |
| 9 | 3- C+ | 2.0 | 90 | 130 | 11- C+ | 2.1 | 90 | 130 |
| 11 | 2- 3+ | 3.2 | 60 | 130 | 10- 11+ | 3.5 | 60 | 130 |
| 12 | 2- 3+ | 4.0 | 60 | 130 | 11- 10+ | 3.0 | 60 | 130 |
| 14* | 3- C+ 2- 1+ | 3.1 1.8 | 60  60 | 125  125 | 10- 11- 9+ | 2.9 | 60 | 125 |
| 15 | 3- C+ | 2.0 | 60 | 130 | 10- C+ | 1.6 | 60 | 130 |
| 16 | 2- C+ | 1.8 | 60 | 130 | 10- C+ | 2.0 | 60 | 130 |
| 17 | 3- C+ | 2.5 | 60 | 130 | 10- C+ | 2.2 | 60 | 130 |
| 18 | 2- C+ | 2.7 | 60 | 130 | 10- C+ | 3.2 | 60 | 130 |
| 19 | 2- C+ | 3.4 | 60 | 125 | 10- C+ | 2.9 | 60 | 125 |
| 20 | 2- 3+ | 2.9 | 60 | 130 | 10- C+ | 1.6 | 60 | 130 |
| 21 | 2- C+ | 1.5 | 60 | 130 | 10- C+ | 1.3 | 60 | 130 |
| 22 | 2- C+ | 3.7 | 60 | 130 | 10- C+ | 3.2 | 60 | 130 |
| 23 | 2- C+ | 2.3 | 60 | 130 | 11- 9+ | 6.0 | 60 | 130 |

C: Generator case. Electrode contact numbers 0-1-2-3 are left sided, contact numbers 8-9-10-11 right sided.. *interleaving stimulation.
